# Characterization of the complete chloroplast genome sequence of *Camellia oleifera* in Hainan, China

**DOI:** 10.1080/23802359.2017.1407687

**Published:** 2017-11-26

**Authors:** Wan Zhang, Yunlin Zhao, Guiyan Yang, Yongcheng Tang, Zhenggang Xu

**Affiliations:** a Hunan Research Center of Engineering Technology for Utilization of Environmental and Resources Plant, Central South University of Forestry and Technology, Changsha, China;; b College of Forestry, Northwest A&F University, Yangling, China

**Keywords:** *Camellia oleifera*, chloroplast genome, next generation sequencing, phylogenetic analysis

## Abstract

*Camellia oleifera*, an evergreen small tree or shrub with high medicinal and ecological values, is mainly distributed in subtropical montane regions of China. In this study, complete chloroplast genome was reported for *Camellia oleifera* in Hainan, China. The results showed that the whole genome was 156,996 bp in length, including a pair of inverted repeats (IRs) of 26,025 bp, a large single copy (LSC) region of 86,649 bp and a small single copy (SSC) region of 18,297 bp. The genome contained a total of 115 different genes, including 81 protein-coding genes, 30 tRNA genes, and four rRNA genes. Among these genes, eight genes contain a single intron and two genes contain two introns. The total GC content of *Camellia oleifera* was 37.29%. The maximum likelihood phylogenetic analysis based on 21 chloroplast genomes showed that *Camellia oleifera* was similar to *Camellia danzaiensis*.

*Camellia oleifera*, an evergreen small tree or shrub of the genus *Camellia* (Theaceae), is mainly distributed in subtropical area of high mountains and hilly terrains in southern China. It is one of the four woody edible oil plants in the world, and is also a unique natural Chinese woody edible oil species (Zhuang 2008; Zhou et al. 2013). Tea oil extracted from *Camellia oleifera* seed has good health care and medicinal value (He et al. 2007). Especially, the unique climate and environmental conditions in Hainan Island gave birth to rich and distinctive oil tea resources (Zheng et al. 2016). However, the seed offspring of *Camellia oleifera* has a complex hereditary, and is easy to mutate, which still exist challenges in the interspecies relationships and phylogenetic(Yang et al. 2013; Huang et al. 2014). Therefore, it is essential to study the chloroplast genome sequence to provide a valuable genomic resource for the future genetic and phylogenetic studies about *Camellia oleifera*. Here, we presented the complete chloroplast genome of *Camellia oleifera* (GenBank accession number: MF541730) based on the Illumina paired-end sequencing data.

The seeds of *Camellia oleifera* were collected from Chengmai, Hainan Province (110.00°E, 19.75°N), and fresh leaves were obtained by seed germination. The specimen was kept in the laboratory at −80 °C under the accession number 20170519YC. Plant Chloroplast Purification Kit (BTN120308) and Column Plant DNA Extraction Kit were combined for extracting Chloroplast DNA. The library was prepared with a NEBNext^®^ Ultra TM DNA Library Prep Kit for Illumina (NEB, Ipswich, MA). The whole-genome sequencing was performed with the 500 bp paired-end sequencing method by the Illumina Hiseq4000 Platform. The raw data were filtered with Trimmomatic v0.32 (Bolger et al. 2014) to get Clean data for subsequent analysis. Protein-coding genes and non-coding RNAs genes were annotated with DOGMA(Wyman et al. 2004) and cpGAVAS (Liu et al. 2012). A circular map of the chloroplast genome was generated with the OGDRAW (Lohse et al. 2007) software.

The chloroplast genome of *Camellia oleifera* is a circular molecule of 156,996 bp in length, with a small single copy (SSC) region of 18,297 bp, a large single copy (LSC) region of 86,649 bp, and a pair of inverted repeats (IRs) regions of 26,025 bp. The total GC content of chloroplast genome is 37.29%, and the corresponding values of LSC, SSC, and IRs are 35.29%, 30.55%, and 42.98%, respectively. It contains 115 different genes, including 81 PCG species, 30 tRNA genes, and four rRNA genes. Among these genes, eight genes (*atpF*, *rpoC1*, *rpl2*× 2, *ndhA*, *ndhB*× 2, *ycf1*) contain a single intron and two genes (*ycf3* and *clpP*) contain two introns.

The evolutionary analyses between *Camellia* species were conducted in MEGA7 (with 1000 bootstrap replicates) (Kumar et al. 2016). The analysis involved 21 amino acid sequences (Figure 1). The phylogenetic analysis showed that *Camellia oleifera* was similar to *Camellia danzaiensis*. The chloroplast genome reported here provided useful genomic resources not only for the exchange of information between nuclear genomes, but also for the population genetics of *Camellia oleifera* and its phylogenetic and evolutionary studies.

**Figure 1.**
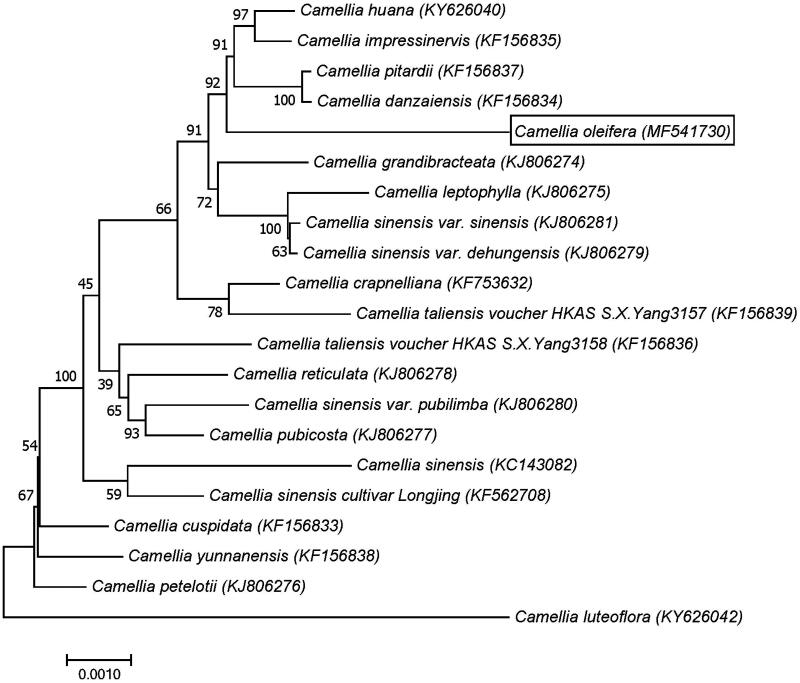
Maximum likelihood phylogenetic tree based on 21 complete chloroplast genomes from *Camellia* family.
